# Motion Noise Changes Directional Interaction between Transparently Moving Stimuli from Repulsion to Attraction

**DOI:** 10.1371/journal.pone.0048649

**Published:** 2012-11-06

**Authors:** Jennifer L. Gaudio, Xin Huang

**Affiliations:** Department of Neuroscience, University of Wisconsin-Madison, Madison, Wisconsin, United States of America; Dalhousie University, Canada

## Abstract

To interpret visual scenes, visual systems need to segment or integrate multiple moving features into distinct objects or surfaces. Previous studies have found that the perceived direction separation between two transparently moving random-dot stimuli is wider than the actual direction separation. This perceptual “direction repulsion” is useful for segmenting overlapping motion vectors. Here we investigate the effects of motion noise on the directional interaction between overlapping moving stimuli. Human subjects viewed two overlapping random-dot patches moving in different directions and judged the direction separation between the two motion vectors. We found that the perceived direction separation progressively changed from wide to narrow as the level of motion noise in the stimuli was increased, showing a switch from direction repulsion to attraction (i.e. smaller than the veridical direction separation). We also found that direction attraction occurred at a wider range of direction separations than direction repulsion. The normalized effects of both direction repulsion and attraction were the strongest near the direction separation of ∼25° and declined as the direction separation further increased. These results support the idea that motion noise prompts motion integration to overcome stimulus ambiguity. Our findings provide new constraints on neural models of motion transparency and segmentation.

## Introduction

Motion transparency refers to the perception of overlapping motion vectors in the same spatial region. Extracting more than one motion vector from a given region is a challenging problem faced by the visual system because spatial cues typically useful for segmentation are absent under this circumstance. Understanding the neural mechanisms underlying motion transparency is important to our understanding of visual motion processing [1]–[4].

“Direction repulsion” is a well known illusion of motion transparency. The perceived direction separation between two overlapping moving stimuli appears wider than the actual separation when the directions of two stimuli are within 90° of each other [4], [5]–[11]. Direction repulsion amplifies the difference between two motion vectors and therefore facilitates the segmentation of transparently moving stimuli.

In contrast to segmentation, integration pools motion signals to overcome local motion ambiguity and to improve signal-to-noise ratio [12]–[16]. Illusions that are linked to segmentation and integration are often found in pairs, reflecting the fundamental and opposing roles of segmentation and integration in visual perception [12]. An example in motion perception is the opposite phenomena of motion contrast [17], [18] and capture [19]. Motion contrast and capture can change from one to another depending on visual stimuli [20]–[22].

Noise in visual images can prompt motion integration (e.g. [23]). However, the impact of motion noise on directional interactions between transparently moving stimuli is not yet clear. It has been found that higher motion coherence is required to perceive motion transparency than to perceive a unidirectional stimulus [24]–[25]. However, when motion noise is present, the perceived direction separation between transparently moving stimuli is not yet known. How does the perceived direction separation change with the level of noise in visual stimuli? Because increasing the level of noise in visual stimuli likely enhances the integration of motion signals, we hypothesize that the perceived direction separation between two overlapping motion vectors decreases as the noise level of visual stimuli is increased. We test this hypothesis in this study and have found that motion noise changes the directional interaction between transparently moving stimuli from repulsion to attraction.

## Materials and Methods

### Ethics Statement

All aspects of this study were in accordance with the principles of the Declaration of Helsinki and approved by the Institutional Review Board at the University of Wisconsin-Madison. Subjects gave written informed consent before participating in the experiments.

### Apparatus

Stimulus presentation and data acquisition were controlled by a real-time data acquisition program (http://www.keck.ucsf.edu/~sruffner/maestro) running under windows XP. Visual stimuli were presented on a 19″ CRT monitor at a viewing distance of 57 cm. Viewing was binocular. Monitor resolution was 1,024×768 pixels and the refresh rate was 100 Hz. Visual stimuli were generated by a Linux workstation using an OpenGL application that communicated with the main experimental control computer over a dedicated Ethernet link. The stimuli were viewed in a dark room with a dim background illumination. A chin rest and a forehead support were used to restrict head movements of the observers.

### Subjects

Four observers participated in this experiment. CC and SW were naïve about the purposes of the experiments. JG and XH are the authors. All observers had normal or corrected-to-normal visual acuity.

### Visual Stimuli

Visual stimuli were two spatially-overlapping random-dot patches presented within a static circular aperture of 7.5° diameter. Each random-dot patch translated in a given direction at a speed of 5°/sec. The dot density of each patch was 3.4 dots/deg^2^ and each dot extended 0.075° (2 pixels). Dot positions were calculated at the accuracy of sub-pixel and rounded to the nearest pixel position. The luminance of the dots and the background were 16.6 cd/m^2^ and 0.08 cd/m^2^, respectively. The two random-dot patches simultaneously moved in two different directions whose vector-averaged direction was 90° (i.e. upward). The motion coherence of each random-dot patch was varied from 50–100%. Motion coherence was controlled by setting a given percentage of the dots (of *one* random-dot patch) to move in a fixed direction and speed while the rest of the dots of that stimulus patch moved randomly in different directions (after [26]). The motion coherences of the two random-dot patches were always the same. The lifetime of the random dot was unlimited (as long as the presentation duration). Once a dot reached the boundary of a viewing aperture, the dot would reappear at the other side of the aperture.

**Figure 1:**
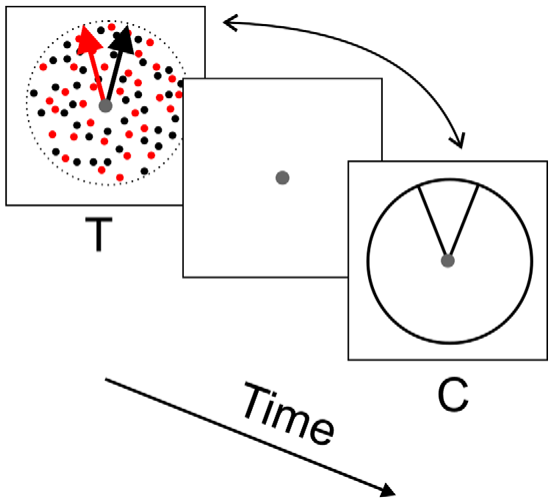
Illustration of visual stimuli and the temporal sequence of an experimental trial. The test stimuli (T) were spatially-overlapping random-dot patches moving in two directions. Red and black colors are used to illustrate two different sets of random dots, whereas the actual random-dot patches were achromatic and had the same luminance. The comparison stimuli (C) were two static lines forming an angle. Subjects compared the direction separation of the test stimuli with the angle of the comparison stimuli. The order of stimuli appearance was randomized.

The perceived separation between the motion directions of the two random-dot patches was measured using “static line-stimuli”. Two lines were presented in a circular outline with a diameter of 7.5°. An angle was formed by these two lines connecting in the center and extending to the edge of the circle. Each line was 3.75° long and 0.04° wide. The angle between the two lines was variable from trial-to-trial. The vector-averaged orientation of the two lines was 90° (see Figure 1 for an illustration of the visual stimuli).

### Procedure

Subjects performed a temporal two-alternative forced-choice (2AFC) task. The overlapping random-dot stimuli and the static line stimuli were shown in two temporal intervals counterbalanced in the order of appearance (Figure 1). The observers compared the perceived angle separation between the moving directions of the two overlapping random-dot stimuli with the static line-stimuli and reported which time interval contained stimuli that had a larger angle separation.

Each trial started with a red fixation spot that was 0.2° across for 500 milliseconds. Subjects were instructed to fixate on the spot throughout the trial. Regardless of the order of presentation, the moving random-dot stimuli were shown for 3 seconds and the static line-stimuli were shown for 1.5 seconds. We chose the duration of the random-dot stimuli to allow for subjects to judge the direction separation at low motion coherences as accurately as possible. During pilot studies, although subjects could perceive transparent motion at shorter durations, a 3-second duration was found to be adequate for subjects to perform the task confidently.

The inter-stimulus-interval (ISI) between the random-dot and the line stimuli was 1 sec. Following the second stimulus interval, the red fixation spot turned white for 1.5 sec during which time subjects pushed one of two buttons to indicate their choice.

We used a staircase method to determine the angle of the line stimuli that matched the perceived direction separation of the moving stimuli. In each staircase, the direction separation between the random-dot moving stimuli was fixed and the angle of the line stimuli was changed adaptively at a step of 3°. The initial line angle was picked randomly within approximately a 20° range wider or narrower than the veridical direction separation. At the end of each trial, if the subject reported the lines as having a wider (or narrower) angle, the following trial presented lines with a 3° narrower (or wider) angle. A “reversal” was scored when the observer switched from reporting the line angle as wider to narrower, or vice versa. The staircase was stopped after seven reversals and the matching angle was determined as the mean of the last four reversals. In the exceptional case when the subject reported down to zero and could no longer make a reversal, the experimenter stopped the staircase and recorded the result of this staircase as zero. On average, 2–3 practice staircases were needed for each condition at the start of the experiment until each subject’s performance stabilized. Over the course of the experiment, all subjects became experienced and consistently demonstrated stable reporting, allowing for the first four staircase sets to be used in calculations. The number of trials in each staircase was variable between subjects and ranged about 15 to 30. It took approximately 2–5 minutes to run one staircase. The testing sequence of the experimental conditions was picked pseudo randomly by the experimenter.

In Experiment 1, subjects reported the perceived direction separation of the overlapping moving stimuli that had a direction separation of 30° or 60°. The motion coherence of the random-dot stimuli was varied from 50% to 100%. In Experiment 2, the motion coherence of the random-dot stimuli was set at either 60% or 100% and the direction separation of the moving stimuli was varied from 15° to 120°.

## Results

**Figure 2:**
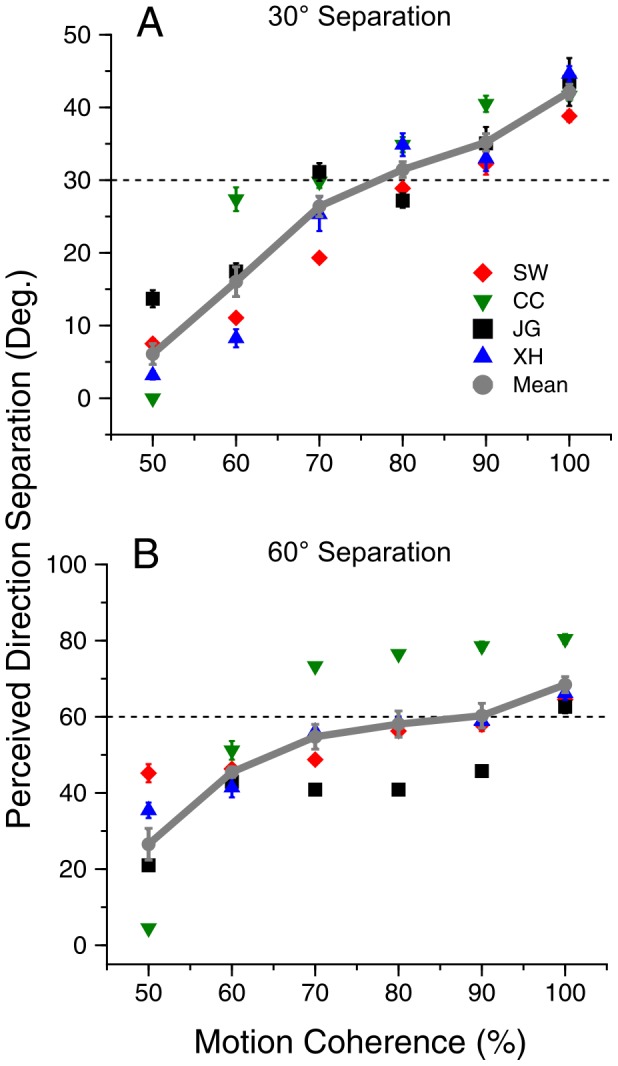
Perceived direction-separation as a function of motion coherence. A. The veridical direction-separation of the random-dot stimuli was 30°, indicated by the dash line. **B.** The veridical direction-separation of the random-dot stimuli was 60°. Error bars represent Standard Errors.

In Experiment 1, we examined the effect of motion coherence on the perceived direction separation between two overlapping moving-stimuli. We found that the perceived direction separation progressively changed from wide to narrow as the motion coherence was lowered (Figure 2). When the direction separation was set at 30° veridically, all subjects could segregate the motion directions of the two overlapping, random-dot stimuli at coherence levels from 60% to 100% and hence perceive transparent motion (Figure 2A). Under these conditions, all subjects reported greater-than-zero direction separations in all staircase trials.

At the motion coherence of 50%, subject CC could no longer see two separate motion directions but instead perceived an integrated single motion direction albeit noisy, while other subjects perceived small direction separations (Figure 2A).

At 100% coherence, the average perceived direction separation across four subjects was 42° (SD = 3.9°) (Figure 2A), showing an effect of direction repulsion as reported previously [5], [6]. The perceived direction separation showed weak repulsion at ∼90% coherence, matched the veridical separation at ∼80% coherence, and was smaller than the veridical separation at the coherences lower than 70% (Figure 2A). At 60% coherence, the average perceived direction separation was 16° (SD = 7.9°), about 47% smaller than the veridical direction-separation. We refer to the perceived direction-separation being smaller than the veridical separation as “direction attraction”. The effect of motion coherence on the perceived direction separation was highly significant (one-way ANOVA, F(5,18) = 24.3, p = 2.3×10^−7^). The progressive shift of the perceived direction separation from repulsion to attraction when the motion coherence was lowered was also found when the veridical direction-separation was at 60° (Figure 2B). At this direction separation, the effect of motion coherence on the perceived direction-separation was also significant (one-way ANOVA, F(5,18) = 5.2, p = 0.0039).

**Figure 3:**
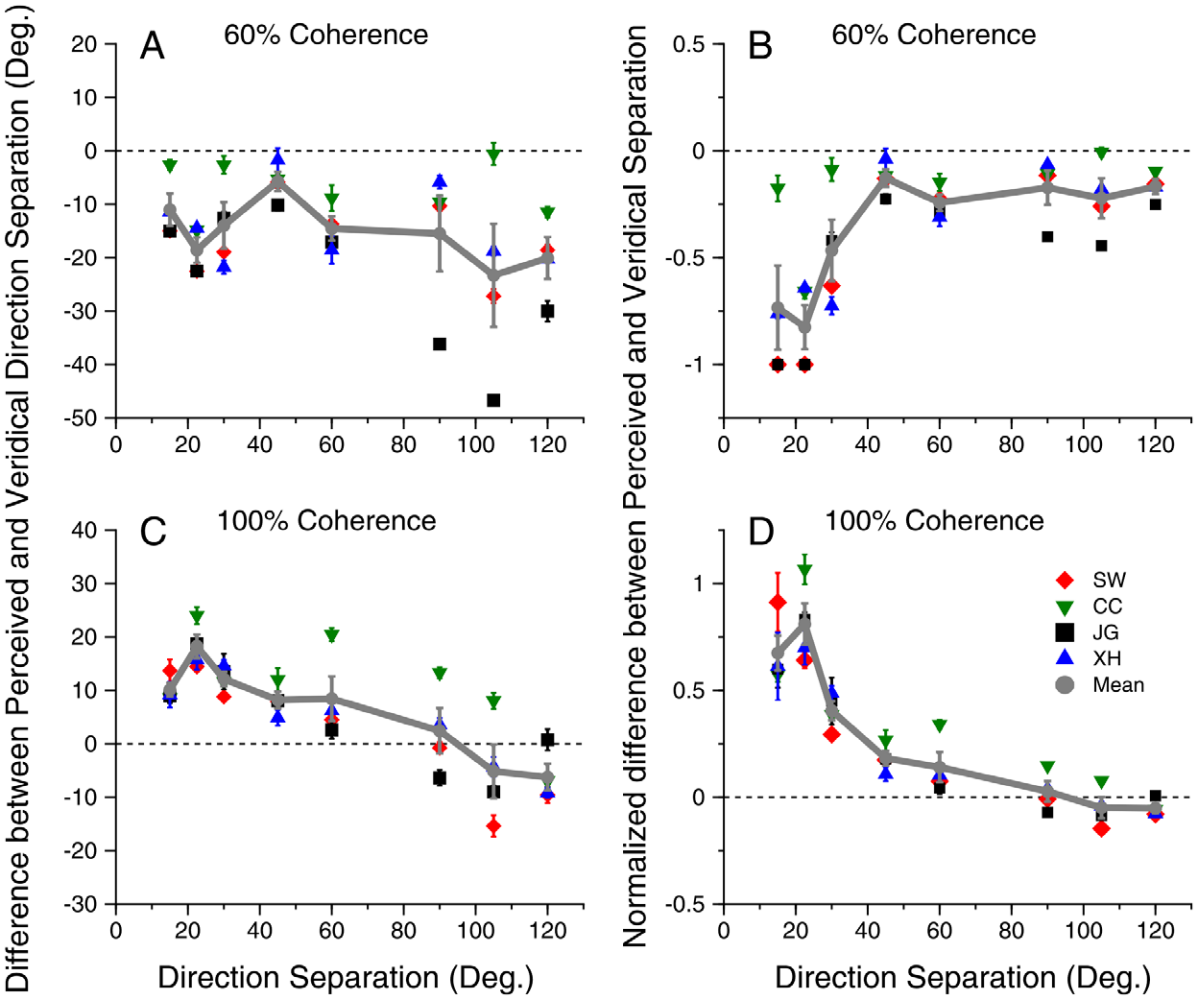
Direction repulsion and attraction as functions of the veridical direction-separation. **A.** Difference between the perceived direction-separation and the veridical direction- separation at the motion coherence of 60%. **B.** Difference between the perceived direction-separation and the veridical direction-separation normalized by the veridical direction-separation at the motion coherence of 60%. **C.D.** The motion coherence was at 100%. Error bars represent Standard Errors.

In Experiment 2, we compared how direction attraction and repulsion depended on the direction separation of the overlapping moving stimuli. At 60% motion coherence, direction attraction occurred across all the direction separations tested from 15° to 120° (Figure 3A). On average, the magnitude of the direction attraction, represented as the difference between the perceived and veridical direction-separations, was about 10°∼20° across all the tested direction separations (Figure 3A). The median magnitude was 14.6° which was significantly different from 0 (signed rank test, p = 7.9×10^−7^). Despite direction attraction, all subjects reported greater-than-zero direction separations in all staircase trials when the direction separation was 30° or greater.

To examine the magnitude of attraction relative to the direction separation, we normalized the magnitude of direction attraction by the veridical direction-separation (Fig. 3B). The magnitude of normalized direction attraction showed a significant effect of dependence on direction separation (one-way ANOVA, F(7,24) = 7.0, p = 1.4×10^−4^). Direction attraction was the strongest at direction separations around 25°, declined as the direction separation increased to 45°, and kept roughly constant at the direction separations larger than 45° (Figure 3B).

To compare the effect that direction separation has on direction attraction with its effect on repulsion, we repeated the experiment with the same group of subjects using a motion coherence of 100%. Consistent with the previous finding [5], [6], the strongest effect of repulsion was reached at direction separations around 25° and declined as the direction separation increased (Figures 3C,D). The effect of direction separation on the magnitude of normalized direction repulsion was significant (one-way ANOVA, F(7,24) = 31.8, p = 1.2×10^−10^).

The normalized effect of direction attraction measured at 60% coherence (Figure 3B) mirrored that of the direction repulsion measured at 100% coherence (Figure 3D). One difference is that, at the direction separations of 90° and larger, the effect of direction repulsion was minimal whereas direction attraction remained at a low level (Figures 3B, D). A two-way repeated measures ANOVA revealed significant main effect of coherence (F(1,3) = 177, p = 9.1×10^−4^), a non-significant effect of direction separation (F(7,21) = 1.9, p = 0.12), and a significant interaction between the two factors (F(7,21) = 27.4, p = 3.4×10^−9^). A non-significant effect of direction separation on the pooled data of repulsion and attraction is consistent with the observation that the normalized magnitude of direction repulsion and attraction roughly mirrored each other and therefore diminished the net effect of direction separation. To directly compare the magnitude of direction attraction and repulsion without the influence of the sign difference, we inverted the normalized magnitude of direction attraction and compared it with the normalized magnitude of direction repulsion. A two-way repeated measures ANOVA showed a significant main effect of direction separation (F(7,21) = 27.4, p = 3.4×10^−9^), a non-significant effect of coherence (F(1,3) = 1.1, p = 0.37), and non-significant interaction between the two factors (F(7,21) = 1.9, p = 0.12). These results suggest that normalized magnitudes of direction attraction and repulsion differ in sign but share a similar trend as the direction separation varies.

## Discussion

### Direction Attraction and Motion Integration

We found that the perceived angular separation between the directions of overlapping stimuli progressively shifted from direction repulsion to attraction as the level of motion noise in the stimuli was increased. When the noise level was high, the perceived direction separation was smaller than the actual separation - the two component directions appeared to “attract” each other.

The progressive, noise-induced shift from direction repulsion to attraction between transparently moving stimuli has not been shown before. In a related study, Braddick and colleagues (2002) [4] found that when the actual separation between motion directions of two overlapping random-dot patches was smaller than 25°, the perceived direction-separation can be smaller than the actual separation (see their Fig. 3C). They indicated that at angles less than 22.5°, subjects were not able to perceive transparent motion; at 22.5° separation, some subjects might see motion transparency while others might not, giving rise to a large individual variation and an average perceived direction separation less than 22.5° [4]. Different from their findings, our results demonstrate the effect of direction attraction is not due to the small direction separation between two moving stimuli, but caused by motion noise. We found that the effect of direction attraction can occur at large direction separations when two motion directions can be simultaneously perceived. Furthermore, our results demonstrate a progressive relationship between the magnitude of direction attraction and the level of motion noise.

Consistent with other studies demonstrating direction repulsion [5]–[7], we did not find a change from repulsion to attraction as the angular separation between two motion directions decreased when each motion component was moving at 100% coherence. As pointed out by Braddick et al. (2002) [4], the discrepancy between their results and others can possibly be explained by the difference of random dot lifetimes. In the study of Braddick et al. (2002) [4], the random dots had limited lifetimes making it harder to segment motion components at small direction separations, whereas in other studies of direction repulsion, including ours, the random dots moved continuously across the viewing aperture when the coherence of each motion component was set at 100%.

Our finding that the perceived direction-separation shifts from repulsion to attraction as the level of noise in the visual stimuli increases is consistent with the idea that stimulus ambiguity can shift the balance between segmentation and integration [12]. More noise in visual stimuli may lead to more integration [13], [23]. This integration functions to suppress noise at a cost of reducing the ability to segment components of overlapping stimuli. Our findings, obtained by varying the level of motion noise, are consistent with previous studies manipulating luminance contrast. Like motion noise, low luminance contrast also causes ambiguity in visual stimuli and may lead to more integration [27], [28]. Using multi-aperture bar or grating stimuli, Kim and Wilson (1996) [27] found that the effect of direction repulsion decreased as the luminance contrast of one of the two motion components was lowered. At the lowest contrast tested, a modest effect of direction attraction was found [27].

One function of motion integration is to pool local motion signals to compute the global motion of the stimuli [13]. When visual stimuli contain two different motion streams, an integrative smoothing operation [14] would reduce the difference between the two motion directions and give rise to direction attraction. Our results showed that motion integration could give rise to a range of perceived direction separations that were smaller than the veridical separation, but the two motion directions could nevertheless be segmented at motion coherence as low as 60% and direction separation equal to or greater than 30°. In other words, a coherent, single-valued percept is not a requirement for the motion integration to occur. As the level of noise in visual stimuli increases, further direction attraction could eventually give rise to the percept of a single motion direction. These features of direction attraction found with random-dot stimuli appear to be distinct from viewing plaid stimuli, in which integration of 1-D motion signals across orientations gives rises to a single, coherent percept of motion, overcoming the aperture problem [29]. Our results emphasize that direction attraction does not occur only at small direction separations and does not always give rise to the percept of a single direction, but instead causes a progressive change in the perceived direction separation as the level of motion noise increases.

### Considerations of Visual Stimuli

We used two static lines forming an angle as the comparison to measure the perceived direction-separation of the overlapping moving stimuli. Our choice of comparing the angular separation of moving directions with the static lines was to encourage subjects to see the directions of both motion components simultaneously to judge the direction separation. The method is similar to that used by Braddick et al. (2002) [4] in their Experiment 2. A concern of using two static lines forming an angle is that an acute line angle may itself appear to be larger than the actual angle. However, this repulsive effect of line angle is small, at about 1∼3° [30], [31]. In comparison, the effects of direction repulsion and attraction found in our study were large, on the order of 10∼20°. The small repulsive effect of the line angle is unlikely to have a large impact on our results.

When the direction of uni-directional stimuli is close to one of the cardinal axes, the perceived direction appears to be repelled from the cardinal direction. This misjudgment of a single direction is referred to as “reference repulsion” and can be as large as 9° [8]. Reference repulsion may contribute to a portion of the direction repulsion measured in the earlier studies [8], [11]. In our experiment, we measured the impact of noise on direction separation using stimuli similar to those used in the classic studies of direction repulsion [5], [6], so that our finding of direction attraction can be directly compared with the earlier results of direction repulsion. Reference repulsion cannot explain the attractive directional interactions found at the low motion coherences.

### Constraints on Neural Models of Motion Transparency

In a population of direction selective neurons, such as those found in the middle-temporal cortex (area MT) in the primates [32], a unidirectional moving stimulus evokes a distributed neural activity. The peak of the population activity is located at neurons whose preferred directions match the stimulus direction. When stimulated by two moving directions that are sufficiently separated, two response peaks would appear in the distributed population responses [33]. Several models have proposed that the two response peaks are repelled away from each other due to mutual inhibition between neurons tuned to each of the stimulus directions [5]–[7]. As a consequence, the repelled response peaks now reside at neurons whose preferred directions are slightly off from the true stimulus directions. Estimating stimulus directions based on the distributed population neural activity would then exaggerate the direction separation between the actual motion directions and give rise to direction repulsion. Our finding helps to constrain models of motion transparency to account for the progressive shift from direction repulsion to attraction as the level of motion noise is increased. Extending the concept in the previous models [7], it may be expected that the two response peaks in the population neural activity become closer to each other when direction attraction occurs at low motion coherence than when direction repulsion occurs at high motion coherence. This prediction can be tested experimentally by recording from direction selective neurons in the visual cortex (e.g. in area MT) of primates while the animals view overlapping stimuli moving in two directions at different coherences.

Raudies and colleagues (2011) [34] recently proposed a model that incorporates center-surround interactions in the velocity domain. Their model can account for direction attraction found at small direction separations by Braddick et al. (2002) [4], as well as direction repulsion. It remains to be determined whether their model can account for our findings of direction attraction caused by motion noise. Generally, our findings of direction attraction induced by motion noise put new constraints on neural models of motion transparency.
